# Hospital Annual Meetings

**Published:** 1919-05-03

**Authors:** 


### HOSPITAL ANNUAL MEETINGS.

### Chelsea Hospital for Women.

At the annual meeting of the Governors the report presented, after referring to the matinée held in March of that year at the Palladium, which added £3,200 to the building fund, stated that this amount, together with that available from the sale of the old hospital, was sufficient to clear off the debt of the new one. There was, however, a debt on the general fund of £4,000, and a considerable amount had to be collected for the building of the Nurses' Home. Until this part of the building scheme was complete half the wards had to be used for housing the nurses and servants. A great effort was now to be made to provide both for the Home and the debt by the Women's Ball to be held at the Albert Hall on May 28. The President (Lord Londonderry) said it was a problem how the funds of the hospitals were to be maintained in the future. There was no doubt the State should interest itself more in the support of hospitals, but it was a most important point that the influences which accompanied private endeavour in this direction should not be weakened if the greater part were taken by the State. It was a matter of congratulation that the people of this nation had come forward as they had done to support these institutions, and this instinct in our race had developed to an extent which was very creditable. It was to be hoped that no idea of State aid would ever supplant the voluntary system.

### City Hospital for Diseases of the Chest.

Presiding over the annual Court of Governors of the City of London Hospital for Diseases of the Chest, Victoria Park, E., held at the Vintners' Hall, Upper Thames Street, E.C., Mr. C. E. Fox, J.P., said that last year's expenditure amounted to £25,098, and was £4,450 more than in 1917. Income from general sources, apart from legacies, showed an increase of nearly £1,000, yet there was a deficit on the year's working of £3,421. In-patients numbered 918, as against 626 a year previously, while the number of out-patients had risen from 8,445 in 1917 to 10,596, with 39,508 attendances last year. The Committee are anxious to proceed with the extension of the institution, which has received the approval of King Edward's Hospital Fund and was postponed owing to the war. Provision is also to be made for research work on a more extensive scale than is at present possible. An almoner's department is also to be established. The Chairman announced that the Duke of Connaught had consented to preside at a dinner to be held in aid of the funds of the institution in November next.

